# Modeling Behavioral Experiment Interaction and Environmental Stimuli for a Synthetic *C. elegans*

**DOI:** 10.3389/fninf.2017.00071

**Published:** 2017-12-08

**Authors:** Andoni Mujika, Peter Leškovský, Roberto Álvarez, Miguel A. Otaduy, Gorka Epelde

**Affiliations:** ^1^Intelligent Transport Systems and Engineering, Vicomtech-ik4, Donostia/San Sebastián, Spain; ^2^eHealth and Biomedical Applications, Vicomtech-ik4, Donostia/San Sebastián, Spain; ^3^IIS Biodonostia, Donostia/San Sebastián, Spain; ^4^Department of Computer Science, Universidad Rey Juan Carlos, Móstoles, Spain

**Keywords:** *C. elegans*, *in-silico* studies, neural simulation, stimuli modeling, behavioral assays

## Abstract

This paper focusses on the simulation of the neural network of the *Caenorhabditis elegans* living organism, and more specifically in the modeling of the stimuli applied within behavioral experiments and the stimuli that is generated in the interaction of the *C. elegans* with the environment. To the best of our knowledge, all efforts regarding stimuli modeling for the *C. elegans*are focused on a single type of stimulus, which is usually tested with a limited subnetwork of the *C. elegans*neural system. In this paper, we follow a different approach where we model a wide-range of different stimuli, with more flexible neural network configurations and simulations in mind. Moreover, we focus on the stimuli sensation by different types of sensory organs or various sensory principles of the neurons. As part of this work, most common stimuli involved in behavioral assays have been modeled. It includes models for mechanical, thermal, chemical, electrical and light stimuli, and for proprioception-related self-sensed information exchange with the neural network. The developed models have been implemented and tested with the hardware-based *Si elegans* simulation platform.

## 1. Introduction

The main aim of simulation of living organisms is the accelerated and controlled testing of different hypotheses on the organism's behavior. This is often necessary when looking for the cause and treatment of an organism's malfunction, either holistically or focusing on its sub-system. *In-silico* solutions provide tools that help to validate them before testing them on real living organisms in *in-vivo* experiments.

The *in-silico* technologies develop toward a complete multi-scale model of the organism, being the ultimate goal of a full virtual organism simulation. Step by step, multi-scale modeling will yield to a complete understanding of all aspects of physiology, from genomes to organs (Walpole et al., 2013).

Developing a complete and a realistic multi-scale model, however, is too complex and hardly feasible at the moment. It is due to the complexity of each organism and the little known interplay of the organism's parts at and between different scales, but also due to its high demand on computational resources that would allow for a viable simulation.

For now, most works focus on individual aspects of physiology and in this paper, the stimulus perception will be elaborated. In this study, the virtual simulation of the *Caenorhabditis elegans* (*C. elegans*) nematode is considered, being one of the simplest organisms with respect to the size of its neural network that consists of 302 neurons. For its relative simplicity but still a rich repertoire of behaviors (mating, escaping from repellents, searching for food, etc.), this tiny worm is widely used for studying the neural activity of living organisms. It is one of the base models in Experimental Biology and Computational Neuroscience when trying to represent its neuronal functions and to reproduce its behavior.

**Figure 1:**
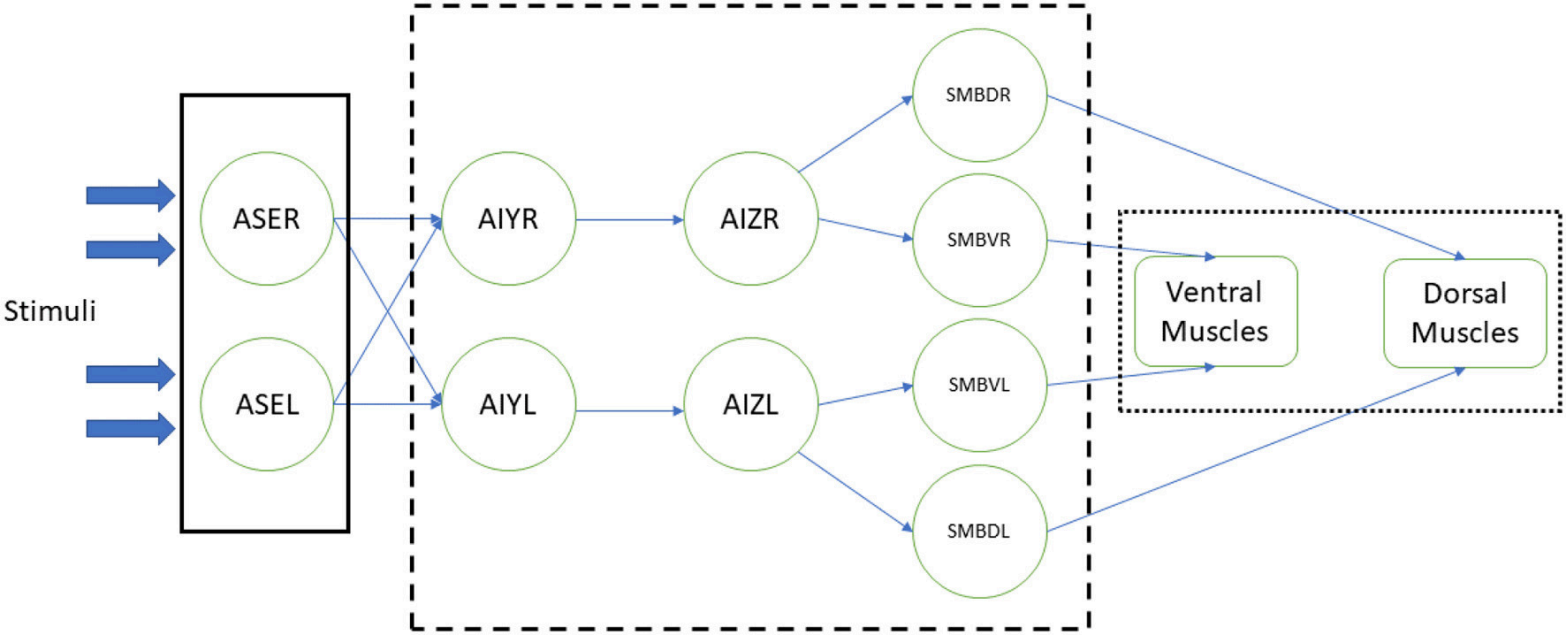
Example of a neural stimulation of the *C. elegans* (Izquierdo and Beer, 2015). Response to chemical sensation was modeled at three stages: the sensory input, the processing of the stimuli by a simplified neural network, actuation of the muscles that result in the worm's motion. Stimulation (plain box) of ASE chemosensory neurons is obtained via an instantaneous function of a derivative operator applied to the recent history of attractant concentrations. Neural circuit (dashed box), with AIY and AIZ interneurons and SMB motorneurons, is connected via sigmoidal synapses. Dorsal and ventral muscles (dotted box) are based in the work of Boyle et al. (2012).

In the related experiments, the modeled somatic neurons are stimulated with some environmental event or change. This stimulus is spread through a reproduction of the worm's neural circuit through which the motorneurons are innervated and the simulated body muscles contracted, making the worm move accordingly. Figure 1 shows an example of such implementation (Izquierdo and Beer, 2015). In this example, the chemosensory neurons ASE (left and right) receive the stimulus from the environment (plain box); this stimulus activates the synthetic neural circuit (dashed box), including interneurons AIY and AIZ and motorneurons SMBD (dorsal) and SMBV (ventral). Finally, the signals that come from the latter make the muscles, in ventral and dorsal part of the worm, contract (dotted box), resulting in the worm's response to the stimulus by approaching or escaping from the chemical substance presented.

Stimuli modeling for the *C. elegans*neural network emulation has followed different approaches. Bryden and Cohen (2008) implement two different types of stimuli, proprioception and mechanosensation. On the one hand, proprioception is implemented making the muscles sense the stretching of their posterior muscle. On the other hand, they consider as mechanosensation the local sensory input that muscles receive in order to stabilize the oscillations of the body. For proprioception, (Boyle et al., 2012) use a similar method, where each segment of the body of the worm takes into account its own stretching level and the state of its posterior segment.

Suzuki et al. (2005a,b) model the response of the nematode to touches at its nose and its tail. Their neural network makes the worm move backward when touched at the nose and forward when touched at the tail. The input signal that is transmitted to the sensory neurons is a stepwise function between 0 and 1.

Reaction to different chemicals is one of the most studied and replicated behaviors. Demin and Vityaev (2014) model the chemical concentration using a negative exponential function (see Equation 1), obtaining a concentration peak at point (*x*_0_, *y*_0_):

(1) $$C(x,y)=e^{-a({\left(x-x_{0}\right)}^{2}+{\left(y-y_{0}\right)}^{2})}$$

Indeed, most of the works that model the *C. elegans*chemosensitive behavior (Lockery et al., 1993; Ferrée et al., 1997; Appleby, 2012) use Gaussian functions to model the chemical concentration, but do not consider other chemosensation assays where the preference of the worm among different chemicals may be investigated. In Pandya et al. (2015), they model a colony of bacteria that creates a chemical concentration. Such concentrations also take a Gaussian shape.

Izquierdo and Beer (2015) also propose a model of the worm that respond to chemotaxis. In this case, two different type of chemical concentrations are used: conical (Equation 2) and Gaussian (Equation 3):

(2) $$C(x,y)=-\alpha \sqrt{{\left(x-x_{0}\right)}^{2}+{\left(y-y_{0}\right)}^{2}}$$

(3) $$C(x,y)=c_{0}e^{-\frac{{\left(x-x_{0}\right)}^{2}+{(y-y_{0}}^{2})}{2\lambda^{2}}}$$

Regarding thermosensation, the neural circuit of Bora et al. (2014) detects gradients in the temperature and the response of the worm is set to follow a certain temperature (not to search the hottest one). The temperature of the environment is modeled with bell shaped functions emerging from different points of the environment.

In general, revised research works do not consider sensilla, branched and long dendrites and transfer the stimulus directly to the neuron, which is considered as a point. Additionally, all efforts regarding *C. elegans*stimuli modeling are specifically focused on one type of stimulus. This can be a drawback when simulating experiments like the ones described in Dusenbery and Barr (1980) and Rankin (1991), where different types of stimuli are combined to analyse the behavior of the animal. Works like Appiah et al. (2016) do make use of different types of stimuli, but do not model what happens in the environment to apply those stimuli, i.e., they apply stimuli directly to sensory neuron without taking into account where the worm is located, its shape or any aspect that could influence the magnitude of the received stimuli.

In this paper, we describe the work done to model and implement a wide range of stimuli for the *C. elegans*nematode, including galvanosensation and photosensation which were not found in the literature. We model stimuli and define their propagation to the neuronal body for further processing. The list of considered interactions is based on the experiments that are defined in Hart (2005), an extensive manual for *C. elegans*behavioral experiments. The stimuli received by neurons were tracked while the worm was crawling in different experiments to assess the correctness of the proposed models.

## 2. Methods

In order to allow for sensory inputs during a neural simulation of the *C. elegans*, external stimuli are generated that are sent to the neural network of the simulated nematode as input for each neuron. From the point of view of behavioral experiments, the following stimuli and corresponding events are considered:

- **Mechanosensation**: gentle touch, harsh touch, collisions with external obstacles and plate-tap;
- **Proprioception**: body curvature and muscle stretch sensing;
- **Chemosensation**: osmotic ring, chemotaxis quadrants with barriers, static point source and dynamic drop test (expansion);
- **Thermosensation**: global temperature change, global temperature gradient, heat point source;
- **Galvanosensation**: electric shock application;
- **Photosensation**: light pulses with local exposure.

In the following, we first explain the mechanisms that are considered for the sensation of external stimuli by the *C. elegans*. These are used for the transmission of the generated stimuli to the respective neurons. Next, we describe the stimuli models that have been used to generate the sensory input for a simulated neural network of the *C. elegans*and indicate its mode of application to the neural network. The sensory inputs result from the interaction of the worm with the environment and self sensing within *in-silico* behavioral experiments. Finally, we describe how these models have been implemented within the *Si elegans* platform; a platform that includes the physical simulation of the worm and its environment as well as the neural simulation of the *C. elegans*, and which provides an easy to use graphical user interfaces (GUIs) for the definition and final visualization of a number of behavioral experiments with a virtual *C. elegans*.

Appendix A in Supplementary Material shows the possible values that each type of stimulus can take. In the following, these values will be discussed without referring to this table again.

### 2.1. Sensory mechanisms

The sensory mechanisms of the *C. elegans*nematode differ for distinct types of external stimuli. Although in some cases the neurons directly sense the external events, often, additional sensory cells are involved which propagate the sensation of the external stimuli from the cuticle to the corresponding neurons. Moreover, and mainly for mechanosensation, the long processes of the receptor neurons also act as dendrites receiving and propagating the considered stimulus.

**Figure 2:**
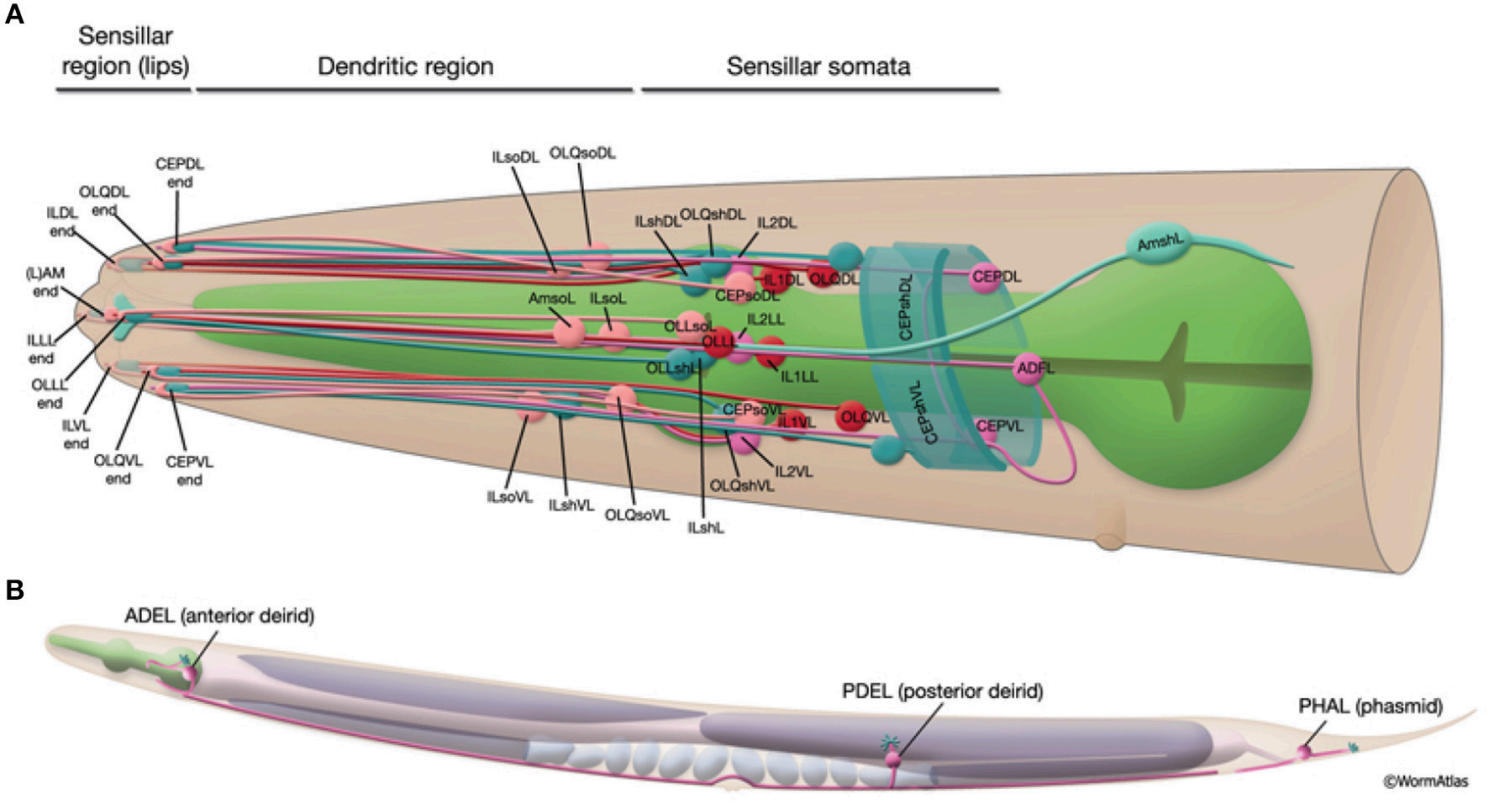
Schematic presentation of the sensillar apparatus of the *C. elegans*. 20 sensilla organs in the head **(A)** and 3 pairs in the posterior to head region **(B)**. Only the left sensilla are shown. The figure has been reproduced with permission from WormAtlas (Altun and Hall, 2010).

**Figure 3:**
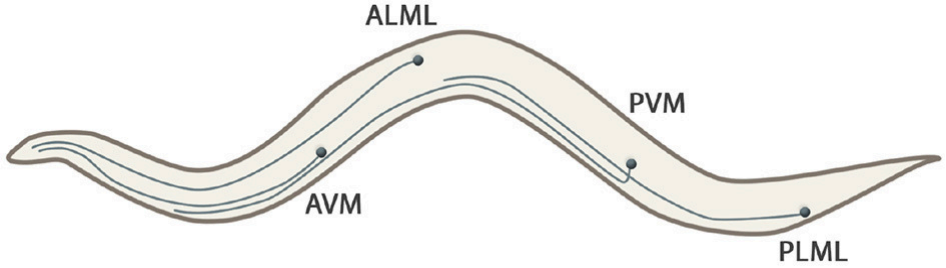
Touch receptors (ALML, ALMR, AVM, PLML, PLMR, and PVM) of the *C. elegans* that extend their processes along the body of the worm. Note that right neurons (ALMR and PLMR) are not represented because they are symmetrical to their left couples.

The following are the types of sensory organs considered for different types of sensory input:

- **Local sensory organs**: Sensilla are simple sensory organs of the *C. elegans* (Altun and Hall, 2010). They are composed of several dendrites of one or more sensory neurons and are surrounded by additional supportive cells. Known are the amphids, the phasmids and the cephalic, inner labial, outer labial and deirid sensilla. In addition to sensilla, several neurons extend their dendrites to the labial or tail section, being not associated with any other sensilla cells. Figure 2 shows the sensillar apparatus of the nematode.
  Considering the specific character of the local sensory organs, the stimuli input sensed by these organs is considered to be hardwired to specific neurons. The specific location and connection of the considered sensory organs is given in Appendix B in Supplementary Material.
- **Long neural processes and branched dendrite trees**: The *C. elegans*nematode has six touch receptor neurons (ALML, ALMR, AVM, PLML, PLMR, and PVM) which extend their processes along the body of the worm. The processes are filled with microtubules, being a unique structure to touch receptors (Goodman, 2006). Figure 3 shows the shape of these mechanosensory long neural processes.
  A 3D model, that will be described in subsection 2.2, of the entire neurons will be considered at the moment of stimulus perception for the related cases.
- **Body wall muscles**: A total of 75 *C. elegans*neurons innervate the 79 body wall muscles posterior to head.
  Considering that the dendritic and the neuromuscular junctions of the motoneurons are connected to distinct muscle cells (see Varshney et al., 2011), possible sensation of stretch in one group of muscles contributes to the stimulation of muscle contractions in the other group. It is hypothesized that the different classes of the motor-neurons coordinate the contraction of the neighboring muscles and thus the forward and backward motion of the worm (Riddle et al., 1997). For the propagation of proprioception stimuli, we consider the neural connections to the muscle cells to be hardwired, similarly to the local sensory organs. We use the connections specified in Varshney et al. (2011), along with the given weights that represent the number of connections formed by the neurons to the respective muscle.
- **Neural soma**: The body of the neuronal cell is the main signal processing unit of the neural network. Here, nucleus and other organelles, both potentially sensitive to external stimuli, are located. For input stimulus cases that are of local influence, such as may be the thermo- or photo-sensation, we consider the body of the neuron to be the principal sensory organ of the corresponding stimuli.

### 2.2. 3D model

To model the sensory capacity of the simulated *C. elegans*as introduced in the previous section (2.1), the location of the sensory organs and neurons of the worm is required. We use the information taken from WormAtlas (Varshney et al., 2011) for the identification of different sensory neurons and organs.

**Figure 4:**
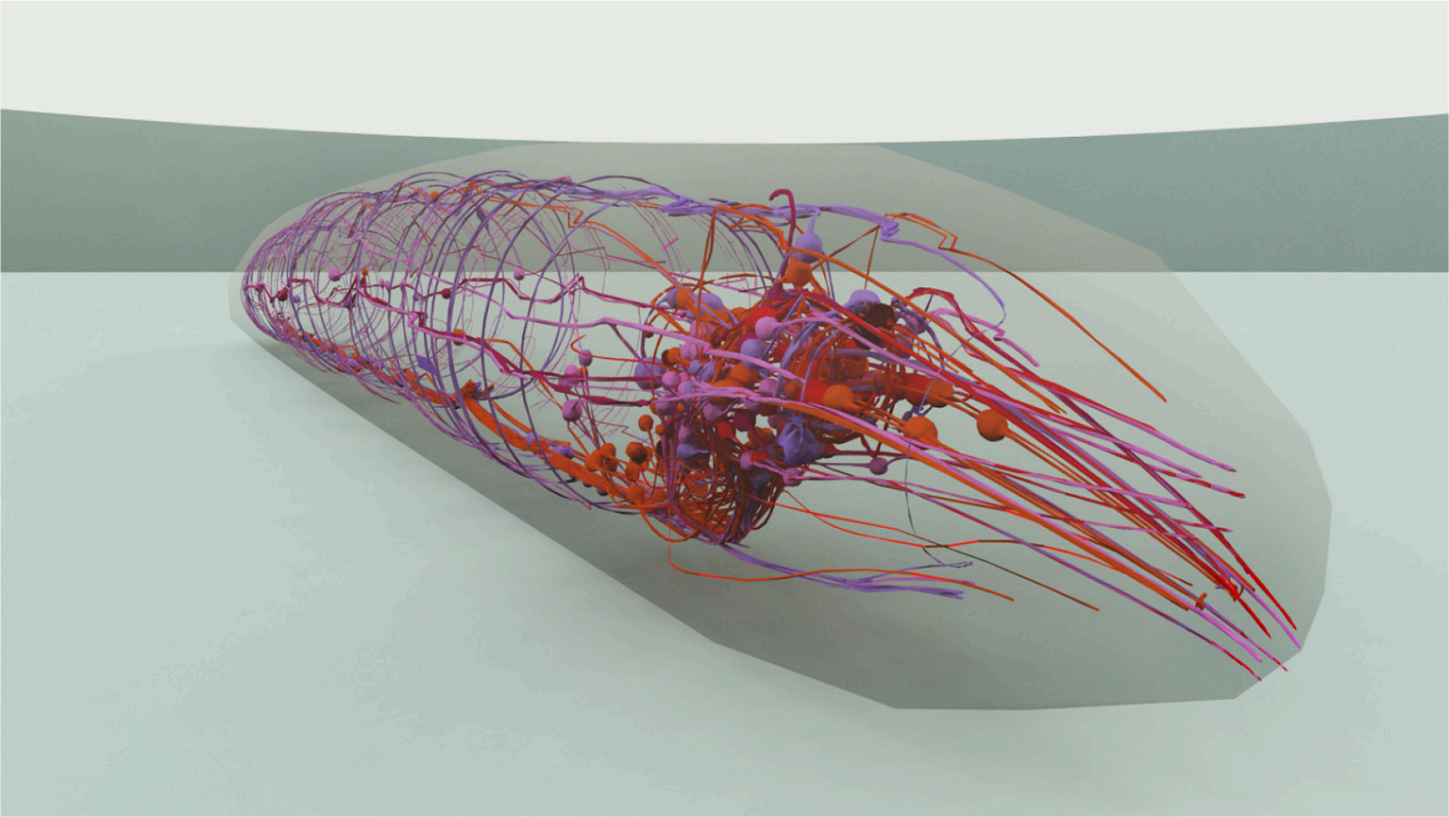
3D representation of the *C. elegans* that has been used for stimuli modeling. The cuticle is shown translucent in order to manifest the neurons and neural network within the worm. A close view on the worm's head is presented.

In majority, the touch sensation experiments require the information on the shape of the neurons and its dendrites in order to modulate the received stimuli with the right exposure level of such neurons. For that, we use the 3D model of the worm constructed in Caltech's Virtual Worm Project (WormBase, 2015). This way, such neurons, as well as their extensions and branched dendrite arbors, are located in three dimensions and stimuli that may occur in their neighborhood can be modulated using the distance from stimuli to the sensory organs. Figure 4 shows the 3D model used, with a view on the head neurons inside the virtual worm.

For the simulation of the locomotion of the the *C. elegans*, a Finite Element Method (FEM) that is based on this 3D model and takes into account the material properties of the muscles and the body is used. The model is composed of 95 body wall muscles divided in 8 longitudinal bundles (all combinations among Left/Right, Dorsal/Ventral and Lateral/Medial). To obtain the model for the simulation, the previously cited 3D model was simplified and tetrahedralized. The cuticle has 330 nodes and each muscle has 24 inner nodes. Each muscle contains 32 tetrahedra and the space between the muscles and the cuticle is composed of 7,340 tetrahedra.

The combination of the signals that come from the motor neurons and activate the muscles (applying the corresponding forces to the simulation nodes that represent muscles) together with other physical aspects of the simulation (material properties, environmental forces) are key to compute the new position (and body shape) of the nematode.

In order to track the curvature of the body of the worm, the 3D representation is divided into 33 slices. The centroids of the slices form a curve that is used to represent the curvature of the worm. This way, the global curvature, i.e., the number of folds or the local curvature at any point can be tracked.

### 2.3. Mechanosensation

Four different types of experiments have been chosen regarding mechanosensation: gentle touch, harsh touch, collisions and plate-tap. In the first three cases external forces are applied at the cuticle of the worm and propagate to the sensory neurons and their dendrites.

Forces of different temporal profiles are applied in each case: soft ramp up and ramp down for gentle touch and stronger gradients for harsh touch. In general, the gentle touch forces should have low amplitude (~10μN) in contrast to the harsh touch, reaching ~100μN (Chalfie et al., 2014). Nevertheless, the amplitude of the force profiles is considered as a parameter to be defined in the implementation. Appendix C in Supplementary Material shows how this ramps have been modeled.

The force profiles for gentle and harsh touch events, *F*_*p*_ are applied at a specific point of the worm, *p*, nevertheless, neurons in the surrounding area are affected. Following the approach by Ohshiro et al. (2011), we use Gaussian spatial smoothing for the distribution of the stimulus to the discrete mesh points of the 3D worm model. The acquired mesh forces are then appropriately propagated to the surrounding sensory neurons. The nuclei of the neurons as well as their dendrites are taken into account. The distances between the mesh points of the cuticle and their nearest point on the 3D model of the neurons (WormBase, 2015) are used to define individual weights for the force propagation. Note that the stress distribution due to surface load is inversely proportional to higher orders of the distance (see e.g., the Boussinesq formula for stress distribution considering inverse proportionality to the square of the distance). However, for current simulation purposes, the simplification to inverse proportionality was made. The suitability of this model is justified in section 3 (see **Figure 9**) where relative differences between the force sensation at different neurons are demonstrated. Finally, individual force contributions at a specific sensory organ are summed up to obtain the final stimulus.

The general force stimuli value received by neuron *N* can thus be expressed by the following equations:

(4) $$F_{c}=\sum_{p}F_{p}Gauss(d(p,c))$$

(5) $$n=\underset{i}{\text{argmin }}\{d(i,c)|i\in N_{3D}\}$$

(6) $$Stimuli_{N}=\sum_{c\in C_{3D}}\frac{1}{d(n,c)}F_{c}$$

**Figure 5:**
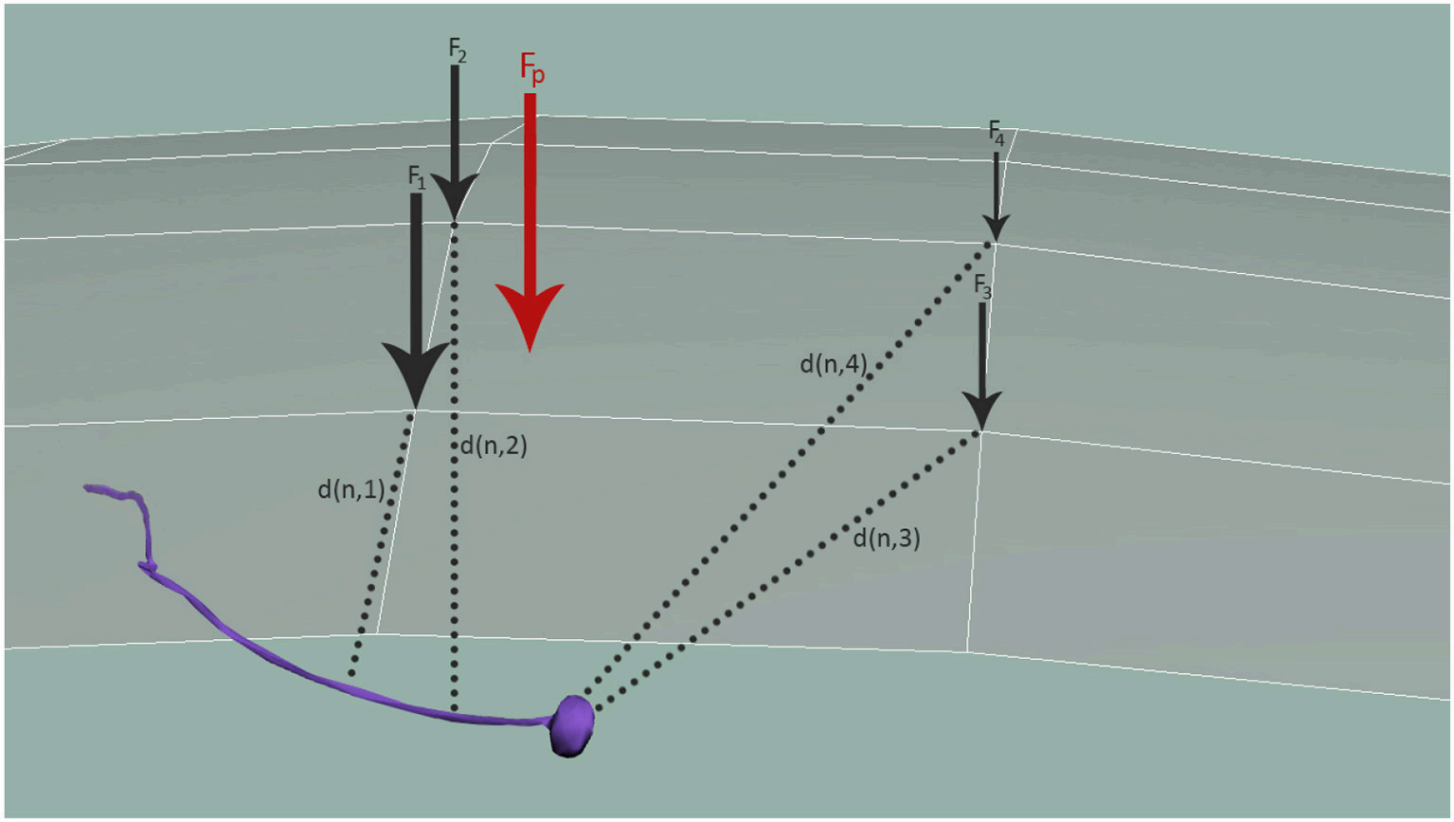
Distribution of mechanosensation forces, *F*_*p*_, among neurons. *F*_*p*_ is distributed to obtain all forces in the simulation nodes of the cuticle, *F*_*c*_. These forces are finally applied to neurons, modulated again with the distance between the neuron and each node, *d*(*n, i*).

In Equations (4–6) *n* marks the node of the considered sensory organ in the 3D model, *N*_3*D*_, being nearest to the cuticle point *c* of the 3D mesh of the cuticle, *C*_3*D*_, and *F*_*c*_ specifies the external load observed at the given cuticle node *c*. The *F*_*c*_ is obtained by spatially distributing the contact forces *F*_*p*_, applied at all the surface points *p* by a Gaussian weighting function $Gauss\left(x\right)=\frac{1}{\sigma \sqrt{2\pi }}e^{-\frac{x^{2}}{2\sigma^{2}}}$, for a predefined variance σ^2^. *d*(*x, y*) is the distance function of the points x and y. Figure 5 shows how *F*_*p*_ is distributed to obtain all mesh forces *F*_*c*_ that are translated to the neuron and modulated by the distance between the neuron and each respective mesh node, *d*(*n, i*).

To model the effect of worm collisions with external obstacles, the point where the collision occurs, *p*, and the magnitude of the force, *F*_*p*_, are computed first and then, the stimulation value of neurons is computed equally to the case of touch events.

Stimuli that emerge in plate-tap are defined in a different way, as we do not directly simulate any shock wave propagation throughout the assay plate. Considering that the plate-tap event involves a non-localized touch stimulus (Wicks and Rankin, 1995), we send a stimulus of a predefined force profile to all mechanosensory neurons. A stimulus of short duration, high gradient (similar to the harsh touch) and selectable amplitude is used.

**Figures 9A,B** show the mechanosensation stimuli recorded in two different experiments, where the location of the touch event is changed angularly and longitudinally, respectively. In the plate tapping case (**Figure 9C**), the bell shaped profile is clearly recognizable in the force profile output. This sensory input is repeated for each mechanosensory neuron.

### 2.4. Chemosensation

There are many different assays that study chemotaxis (Hart, 2005). Some of these experiments use a plate divided into 4 quadrants with different substances, and several worms are placed in the center of the plate. Then, the percentage of worms that go to each quadrant is analyzed. In other assays, a point of a different substance is placed on the agar or a drop of a toxic substance is diluted in the environment where the *C. elegans*is found. The osmotic ring consists of a chemical arranged in a ring central to the assay plate.

The following substances can be considered for the experiments described above:

- **Attractant:** NaCl, biotin, ethanol, butanone.
- **Barrier:** CuSO4, SDS.
- **Repellent:** quinine, benzaldehyde, diacetyl.
- **Immobilization of worms:** Sodium azide.

It is assumed that the chemical substances of the osmotic ring and the chemosensation quadrants experiments, considering as well the chemosensation barriers and the static point source, do not diffuse into the surrounding plate substrate during the assay. Nevertheless, the static chemical point source is modeled as a solution to a steady state where a constant concentration is assumed at the defined source point and is dispersed onto the assay dish. This setting allows for definition of circular gradients of volatile attractants or repellents, which can be sensed by the worm at a distance. For that, we use an approximation of the chemical concentration by Gaussian gradients, similarly to Izquierdo and Beer (2015):

(7) $$c=c_{0}e^{-\frac{{\left(x-x_{0}\right)}^{2}+{\left(y-y_{0}\right)}^{2}}{2\lambda_{c}^{2}}}$$

In Equation (7), for *c*_0_ expressing the steady point source concentration, λ_*c*_ controlling the diffusivity of the substance and *x*,*y* representing the relative spatial position on the assay dish to the point source.

For three experiment types described above, during the experiment, the position and the shape of the whole body of the worm is computed and thus, the positions of all chemosensory neurons and organs are known at any moment. Then, the concentration of each substance at this location is transferred to each neuron as a chemosensory stimulus.

The dynamic drop test represents a dry drop test behavioral experiment, where a drop of chemicals is delivered in the vicinity of the worm (typically 0.5–1 mm) (Hart, 2005) and is let to diffuse within the agar. Usually a position anterior to the nose or posterior to the tail is selected in order to test the different sensing of the nematode by amphids or phasmids neurons. Due to absorption and diffusion of the chemical in the agar and due to the motion of the worm, the worm's sensory organs start to notice an increasing concentration of the chemical substrate and initiate attraction or avoidance behavior via the associated sensory neurons. We model the chemical diffusion following the diffusion equation in 2D:

(8) $$\frac{\delta C(x,y,t)}{\delta t}=D\nabla^{2}C(x,y,t)$$

In Equation (8), for constant diffusion coefficient *D*, expressing the concentration *C* at point (*x, y*) in time *t*. This is also known as the heat equation, which, in the isotropic case can be solved by Gaussian blurring.

Finally, the absolute molar concentration, i.e., the density of the chemical substance as calculated at the specific point on the 2D assay dish, is sensed at the location of each sensilla (i.e., sensory organs) and is then distributed to the neurons according to the observed neural connections. Although currently mainly the amphids, inner labial sensilla and phasmids are reported as chemosensory, for completeness, we consider and propagate the chemical concentrations also to the rest of the sensory organs (deirids, outer labial sensilla and cephalic sensilla).

### 2.5. Thermosensation

For thermosensation, three types of experiments have been modeled: global temperature change in time, temperature gradient in space and local heating.

The global temperature change defines a general increase or decrease of the temperature in the simulated environment and the stimuli received by the worm are not dependant on the position of the worm in the plate. The temperature value of the simulated external environment, generally being between 15 and 25°C, is directly reported to the thermosensory organs. For the inner neurons, it is not considered that the body of the worm may insulate, disperse, transport or in any other way influence the temperature sensed. **Figure 9D** shows an example of such experiment in which the base environmental temperature is set to 20°C which is gradually changed in time from 15 to 25°C. After the end of the event, the environment temperature keeps the last defined value.

The two following experiments depend on the current position of the worm: global temperature gradient and local heating, which define linear and circular gradients, respectively. Although the position of the worm is dynamic and may affect on the thermal properties of the environment, the gradient types are considered as a solution to steady state of the heat equation for continuously heated left-to-right and point sources, and are thus not updated in time.

In a similar way to the chemosensation case, the position of thermosensory organs is computed at any time and the temperature at this point is passed to that neuron as stimulus. In case of the intensively branched neurons PVD and FLP, which cover the entire body of the worm, only the minimal and maximal temperature, respectively for PVD and FLP, as being sensed close to any part of their dendritic arbour, will be transmitted. This is due to the fact that the PVD neurons are known to respond to acute cold shock, while the FLP for noxiously high temperatures.

### 2.6. Galvanosensation

In *in-vivo* galvanosensation experimental assays, electric pulses of predefined profiles and customisable amplitude, duration and repetitions, are typically applied to the whole body of the nematode since directed charge to specific neuron is hard to achieve (Shanmugam, 2017). We model galvanosensation experiment applying electric pulses directly to all the neurons of the simulated worm. A box function is used for a single electric pulse, i.e., a function that alternates 0 values and fixed values. Adding shock frequency and shock duration, finally a model that can be considered as a spike train is used to generate the stimulus input for the neurons. In **Figure 9E**, a train of electrical impulses with magnitude 10 nA, frequency of 10 Hz and pulse duration of 20 ms is tranferred to all neurons. A box function allows for applying short pulses, modeling thus spikes of neural communication or constant currents like in Shanmugam (2017).

### 2.7. Photosensation

*Caenorhabditis elegans* reacts to flashes in its head and its tail reversing its locomotion, i.e., a flash in the head when moving forward makes the worm to stop and to move backward and vice versa (Ward et al., 2008). Moreover, the acceleration magnitude depends on the wavelength of the light. During photosensation experiments, light pulses of customisable wavelength, amplitude and duration are applied directly to desired parts of the worm and the neurons residing in them. A circular light beam being applied at specific parts of the body is defined. The neurons which fall into the illuminated area, receive the photosensation stimulus. A box profile of a given width is applied to simulate a constant intensity within the whole beam, as would correspond to illumination by a laser pointer. It is assumed that only neurons are receptive to this sensory input and that the rest of the body does not absorb the light and thus does not attenuate or alter in any form the simulated light beam and its intensity.

In contrast to galvanosensation, the stimulus is defined to be constant during the whole period of the stimulus and not alternating between 0 and the maximum amplitude, i.e., it is a unique box function and not a sequence of them. In **Figure 9F**, the resulting stimuli input for a photosensation experiment is shown. A number of light impulse events, with increasing magnitude, is applied at different locations from the head to the tail. The beam radius is set to 0.2 mm and duration to 190 ms. The stimuli input as registered by different neurons (OLQDR, AVFR, AS4, VA8, PVPL, and PLMR), with their soma located at different positions along the body of the animal, is shown. Note that the different magnitudes have been used to better visualize the separate events. It can be seen that the selected neurons correctly receive the light pulse stimulus that depends on their position.

### 2.8. Proprioception

Two types of proprioceptive stimuli have been defined as a means to provide the neural system of the nematode with self sensing information: local curvature (Wen et al., 2012) and muscle stretching (Boyle et al., 2012).

**Figure 6:**
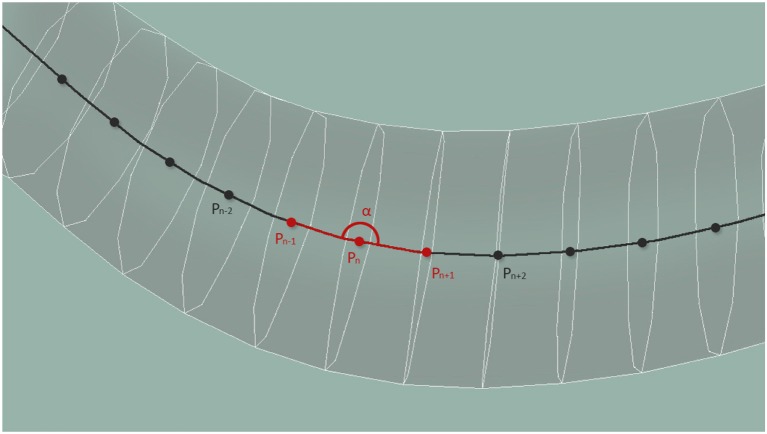
Central points located along the whole body of the worm are used to compute the curvature of the body at that point.

According to Wen et al. (2012), local curvature is key for the locomotion of the worm. We measure the curvature property of the worm along its centerline at evenly distributed 33 points. Locally, the curvature is estimated from 3 central point neighbors, and is limited to a planar curve corresponding to the surface plane of the plate. For each neuron, the value at the central point nearest to the nucleus of the neuron is transmitted. In case of multiple sensory points, as is the case of the long processes of some mechanosensory neurons, or the elongated axons of the A- and B-type motor neurons, the amplitude of the response is normalized by the number of stretch sensing segments. Figure 6 shows the computation of the curvature, α, using central points located along the whole body of the worm.

For the muscle stretch related proprioception, stimuli are distributed to the neighboring neurons. In case of the muscle stretch sensing the stimulus is send directly to the motor-neurons which synapse to the respective muscles. This is due to the fixed connections formed between the muscle cells and the motor-neurons. In Boyle et al. (2012), additionally a gradient decreasing linearly form head to tail is applied to modulate the amplitude of the stretch stimuli and account thus for diminishing muscle efficacy of the worm. In our case, we consider such compensation to be a property of the sensitivity of each neuron and thus it should be considered during the specification of the neural models by the end user.

### 2.9. Implementation

**Figure 7:**
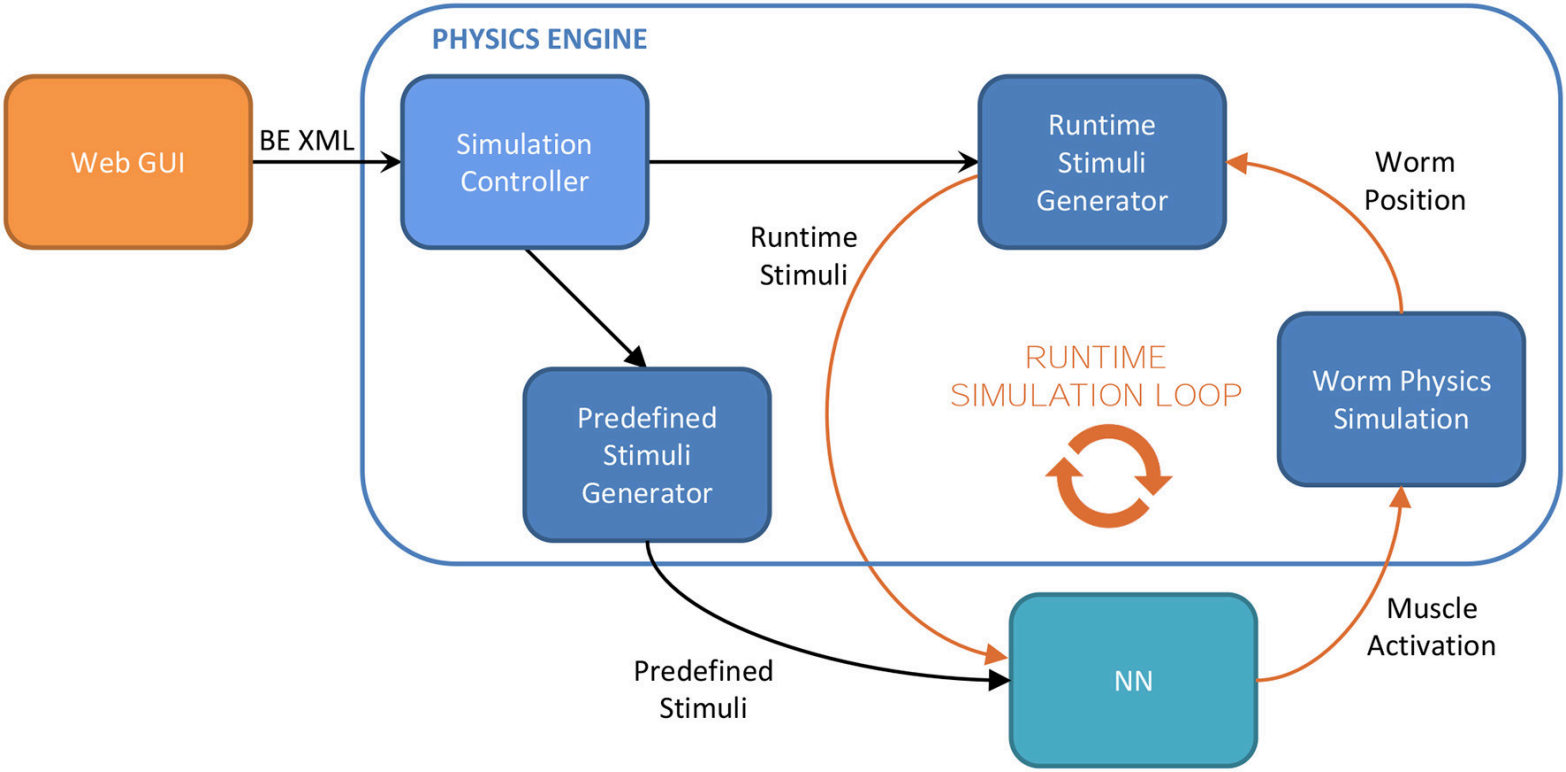
Overview of the simulation workflow of the *Si elegans* platform, with depicted modules of the stimuli generation.

Stimuli models that have been defined in previous sections have been implemented for the *Si elegans* platform (Blau et al., 2014). This platform provides a Neural Network (NN) that replicates the complete Biological Nervous System (BNS) of the *C. elegans*and a Physics Engine (PE) that simulates the emerging physical behavior. In this section, we describe the workflow of the *Si elegans* system regarding the generation of stimuli for the NN. The related processing flow is shown in Figure 7.

Access to the *Si elegans* platform is available to the end users via web. Making use of a GUI, the user defines all the aspects of the *in silico* experiment that will be carried out in the system. Mainly, the neuron models that will be implemented in the NN and the parameters that specify the interaction to be considered for a given behavioral experiment. Once the experiment is simulated in the loop that connects the NN and the PE, the results are brought back to the web and displayed using another GUI that makes use of a virtual representation of the nematode and graphs to show them.

**Figure 8:**
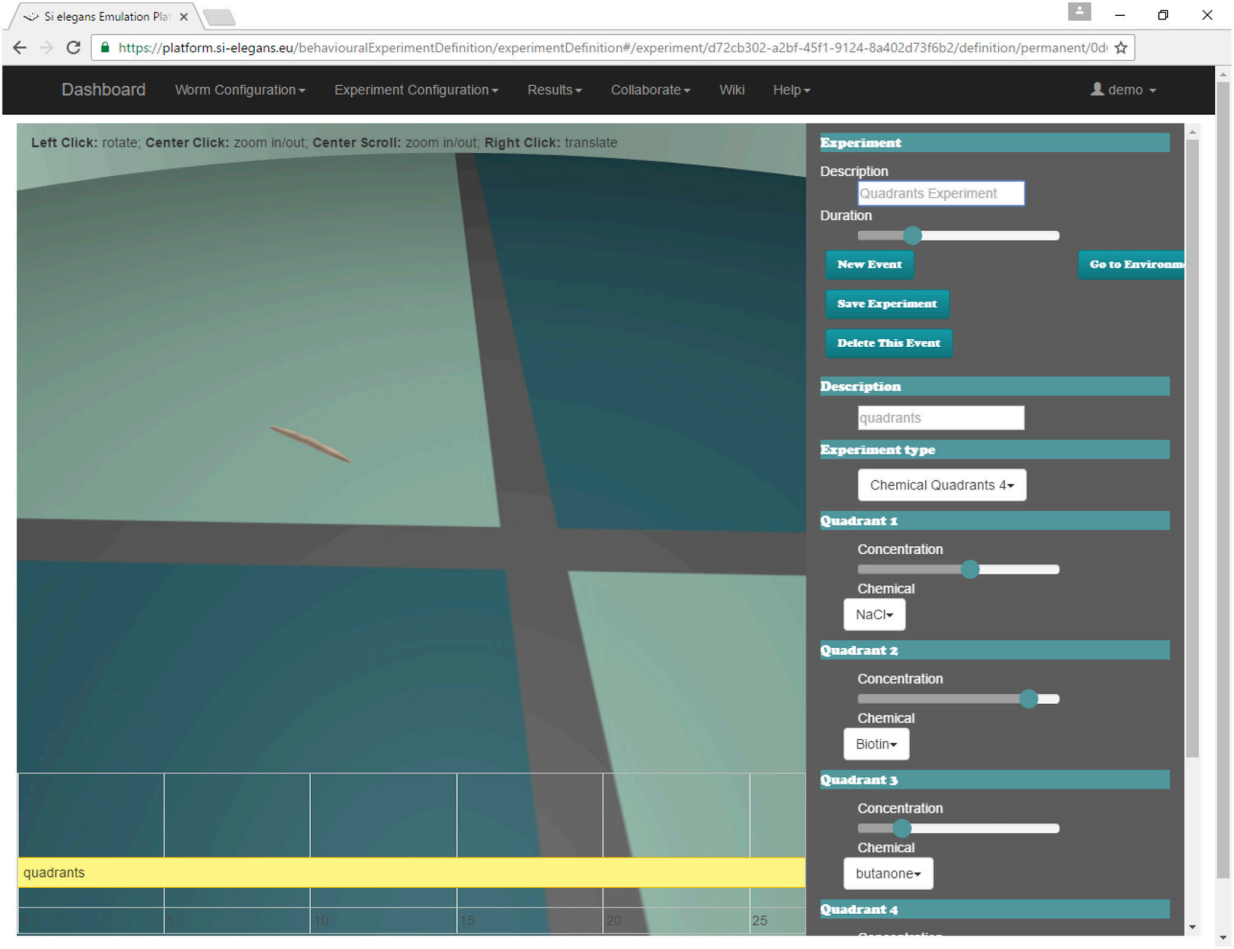
Chemosensation quadrants behavioral experiment being defined in the web GUI tool.

The user may define the experiment that will be simulated by the system through the web GUI (Mujika et al., 2016). Figure 8 shows the interface of the web. A 3D reproduction of the worm shows some aspects of the experiment to be simulated (position and orientation of the worm, chemical application point…); the timeline at the bottom shows the events that are scheduled during the experiment (punctual events such as touching the nematode or permanent events like osmotic rings); and the menu in the right side of the GUI is used to change all parameters of the assay.

Once the experiment is completely defined, all the information about the experiment is codified in a Behavioral Experiment (BE) XML and sent to the PE. Epelde et al. (2015) explains how most common *C. elegans*behavioral experiments (Hart, 2005) can be encoded in an standard XML.

In principle, we do not discern between various sensory capabilities of each neuron. Potentially, all stimuli can be sent as input to all neurons. As such, and although in reality it is not very probable, one neuron can be sensitive to all chemicals, mechanical inputs, proprioception stimuli, thermo-stimuli, etc.

The main criterion for stimuli propagation to the neurons is the spatial distance to the stimuli application point and the area of influence of each concrete stimulus. Due to the existence of specific sensory organs of the *C. elegans*, like sensilla or muscles, which are directly linked to a given sensory neuron, these direct connections are considered within the PE. All types of stimuli are computed and transferred separately and transferred separately to the neurons. Finally, it is up to the implementation of the neuron model to decide whether to consider all or ignore some of the stimuli inputs obtained.

A stimulus can be computed in different stages of the simulation process. Some types of stimuli, denoted as predefined stimuli in the following, such as touching the worm in its nose, can be processed regardless of the position or the shape of the worm at that point. To compute other types of stimuli, runtime stimuli, such as collisions with elements in the environment, current information about the location and shape of the worm is needed.

The predefined stimuli computation happens before the SC starts the simulation and the result is also transferred to the NN before the simulation. Runtime stimuli generation happens during the simulation, working in a loop beside the worm physics simulation and the NN. At each simulation timestep, the position of the worm and its shape is computed activating its muscles with the signals that come from NN. This information, combined with the one that comes from the BE XML, is used to compute the remaining stimuli and transfer it to the NN for the next timestep.

Therefore, implemented stimuli types can be divide in two groups:

- **Predefined stimuli:** Gentle/Harsh touch, plate-tap, global temperature change, electric shock application and light pulse exposure.
- **Runtime stimuli:** Collisions with obstacles, body curvature sensing, muscle stretch sensing, osmotic ring chemosensation quadrants, chemical static point source, chemical dynamic drop test, global temperature gradient, and heat point source.

This separation into two types of stimuli is made in order to achieve an efficient simulation. All the computations that can be done in previous stages are taken off from the simulation loop.

For example, in mechanosensation stimuli generation, considering that a steady 3D model is used, the force distribution weights of the Equation (4) as well as the force propagation weights of the equation 6 can be pre-computed prior to the simulation. Also, to speed up of the calculations, we zero the values of the Gaussian function below a certain threshold.

Also regarding mechanosensation, stimuli that are generated due to experiments where the worm is touched by an external tool (a Von Frey hair, for instance) can be computed offline, but collision with obstacles cannot be foreseen. That is why collision forces are simulated by the physical engine during the simulation (Mujika et al., 2014).

Stimuli generated in chemosensation and thermosensation experiments are generated in runtime, because the position of the worm is needed to know which chemical, and in which temperature, is surrounding it. The only exception is global temperature change, since in this experiment, it is considered that temperature is the same in the whole environment of the worm.

Stimuli that occur in galvanosensation and photosensation assays are computed before the simulation starts, because it is considered that laser and electric pulses are applied to the worm regardless of its position.

## 3. Experiments and results

In order to confirm the correctness of generated sensory input, several tests have been carried out. First, we analyse the predefined stimuli generated in touching events in order to validate the spreading model described in the previous section. After that, we focus on runtime stimuli, i.e., those that are generated during the simulation and take into account the position of the *C. elegans*. Since the real magnitude of the received stimuli by the neurons of the worm cannot be tracked by current technologies, we focus on analyzing basic coherence of the generated signals. Note that an ultimate validation of the realism of the behavior of the nematode depends on a full and realistic set-up of the neural network and the neural models, being part of open research, that is currently not available, and is therefore out of scope of this paper.

**Figure 9:**
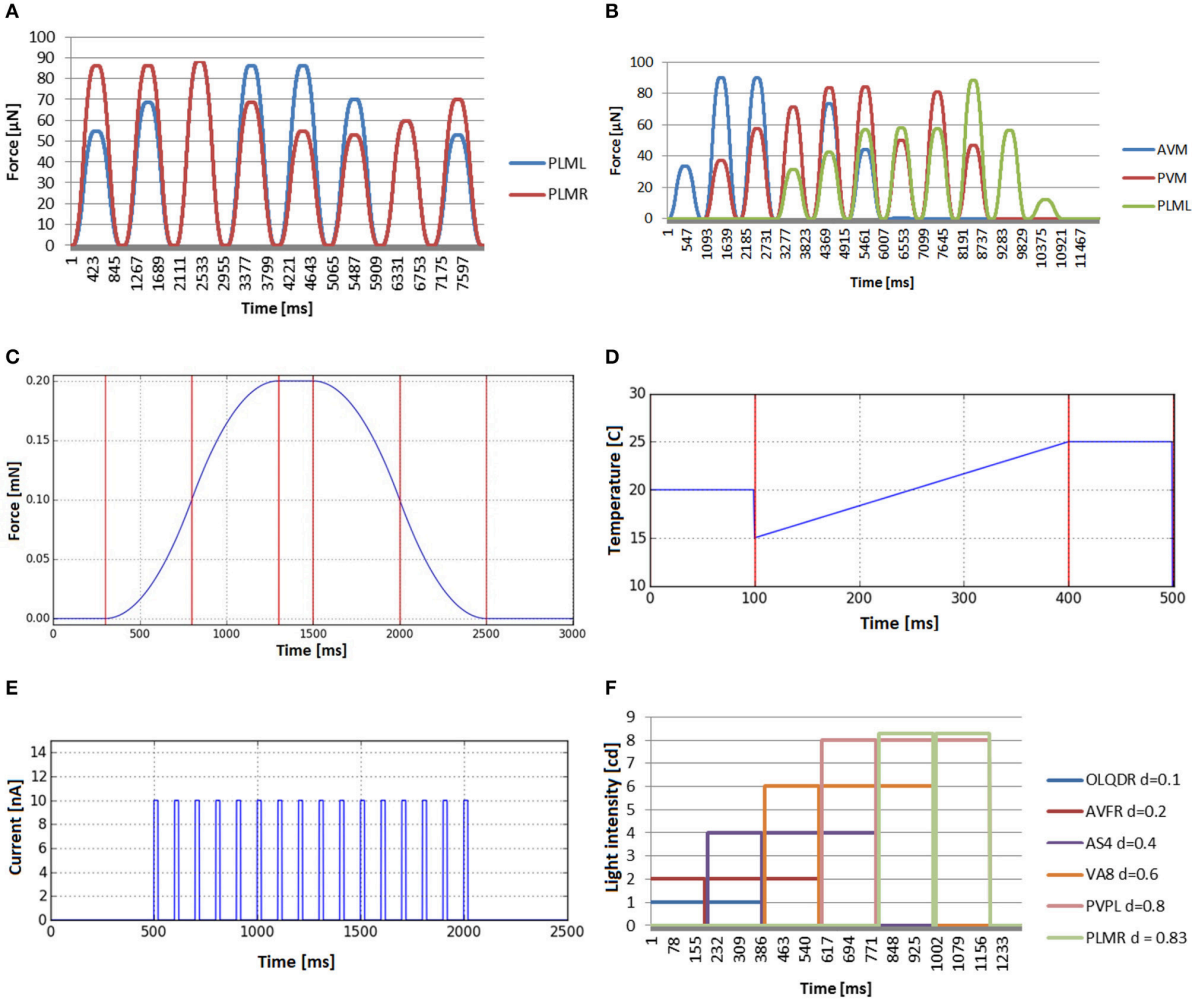
Stimuli that can be computed before simulation starts, because they do not depend on the position of the *C. elegans*. **(A)** Mechanosensation in the tail (neurons PLML and PLMR), changing the application angle (from top of the worm going around the transversal plane in counter-wise manner). **(B)** Stimuli received by three neurons that are located in different lenths of the nematode (AVM, PVM and PLML) when the worm is touched along the body. **(C)** Bell-shaped stimulus received by any mechanosensory neuron when tapping the plate that contains the *C. elegans*. **(D)** Global temperature change received by any thermosensory neuron. Default temperature is set to 20°C. Temperature changes in time from 15 to 25°C. After that, the environment temperature keeps the last defined value. **(E)** Electrical charge stimulus transferred to all neurons in the form of a train of electrical impulses. **(F)** Stimuli input registered by neurons located at different lengths (OLQDR, AVFR, AS4, VA8, PVPL, and PLMR) when light impulse events, with increasing magnitude, are applied at different locations from the head to the tail.

For touching event validation, several touch events were applied at the tail and the ventral side of the worm. In the first one, the touch event is applied at the same length (the tail), but changes the application angle (starting from top of the worm going around the transversal plane in counter-wise manner). Mechanosensory neurons that are placed at different angles are tracked (PLML and PLMR). We see in Figure 9A how the stimulus evolves in a similar way but with delay in each neuron, i.e., when the touching event approaches the neuron of one side, the signal transmitted to such neuron increases and the opposite signal decreases. In the second case (Figure 9B), touch events are spread along the body. Three neurons have been selected to evaluate the stimuli generated. Such neurons have mechanoreceptive processes distributed along different parts of the body: anterior half (AVM), middle body (PVM), and posterior part (PLML). The different force magnitudes, observable in the graph plots, correspond to the different sensation regions of the inspected neurons. The forces transferred to AVM reach their highest value near the head and disappear at the tail, while in the case of PLML, the opposite occurs. The forces received by PVM disappear only at the extremes of the body and are clearly higher in the central part of the body. The obtained results confirm a plausible distribution of the mechanosensory stimuli.

To test the correctness of different stimuli generated during the simulation in the PE, the locomotion of the worm was induced by a Central Pattern Generator (CPG) model and stimuli values received by specific neurons were recorded. Four different tests were carried out: chemical quadrants, chemical drop, thermosensation linear gradient and proprioception test.

For chemosensation, two different assays have been done. In the first one, *C. elegans*crawls and accelerates in a plate divided in four different chemical quadrants (Figure 8). The quadrants are divided by a barrier made of a different chemical. Specifically, the animal starts in one of the quadrants (with Biotin at 44 mM of concentration), crosses the barrier (with NaCl at 20 mM of concentration) and arrives at another quadrant (with ethanol at 60 mM of concentration).

**Figure 10:**
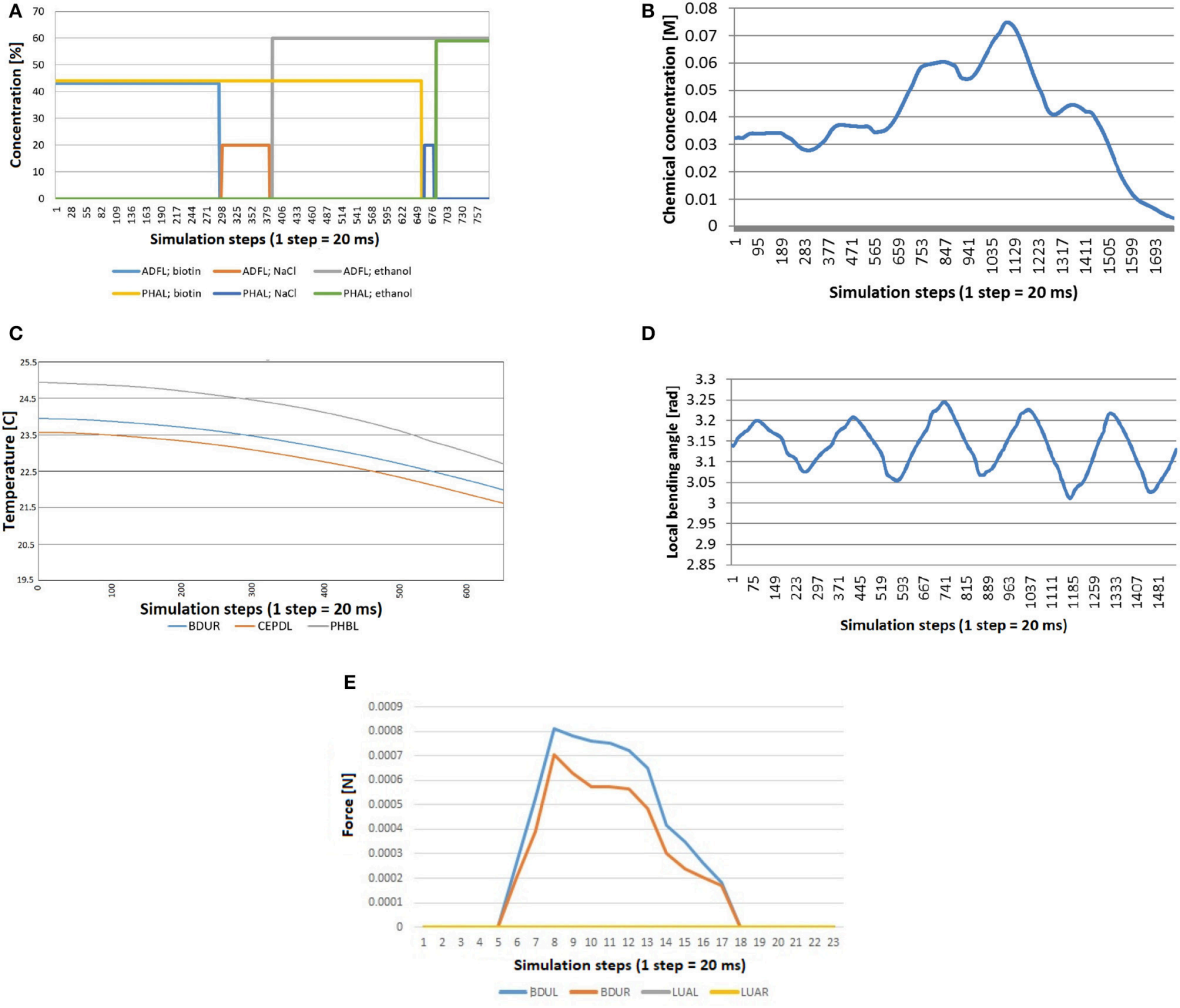
Stimuli computed during simulation, because they depend on the position and the shape of the worm at each moment. **(A)** Quadrants assay: the worm crosses quadrants and barriers and consequently neurons receive concentration of different chemicals. **(B)** Chemosensation received by the neuron IL1R when the worm crawls across a gradient generated with a chemical drop. **(C)** Thermosensation stimulus in BDUR, CEPDL, and PHBL when the *C. elegans*crosses a lineal thermal gradient. **(D)** Proprioception stimulus in AS3 when the locomotion of the nematode is activated with a CPG. **(E)** Mechanosensation induced by collision between an obstacle and the head of the worm. Neurons in the head (BDUL and BDUR) are activated and the stimulus was not received by the neurons that are not located in the head (LUAL and LUAR).

Figure 10A shows the stimuli transferred to neurons ADFL (in the head of the worm) and PHAL (in the tail). As expected, every line is 0 during the whole experiment except for the time the worm is in a specific quadrant. At this moment, the signal is equal to the concentration of that chemical in that quadrant. Specifically, the sequence of the concentration sensed by the neurons is: biotin for ADFL and PHAL; NaCl barrier for ADFL and biotin for PHAL; etc. From the plot it can be seen that the chemical sensing in ADFL precedes those sensed in PHAL that is due to the positioning of the neurons (located in head and tail, respectively) as well as the motion of the worm induced from the biotin region toward the ethanol region. The speedup of the changes in the second half (i.e., entering the ethanol region) are attributed to the acceleration of the worm's motion that stabilizes after a while since the CPG initiation.

In the second test for chemosensation, the nematode crosses a gradient generated by a drop of chemical. Figure 10B shows the stimulus received by the neuron IL1R during such experiment. A combination of a sinusoidal pattern and a Gaussian-like curve function can be observed. The sinusoidal pattern is due to the locomotion of the worm and the Gaussian-like curve represents how the worm first approaches the source of the stimulus and moves away after that. Again, the acceleration of the worm can be observed in the faster decrease of the signal.

To test thermosensation, a linear gradient was set in the plate where *C. elegans*crawls. The temperature in one end was 20 and 25°C in the other end and the worm crawled toward the coolest part. In Figure 10C, we see that the computed thermal stimuli in neurons BDUR, CEPDL, and PHBL decreases as the animal advances. The velocity of the worm was increased during the experiment to check that temperature decreases in a similar way.

Regarding proprioception, the CPG was activated in a worm isolated from any other force (friction or gravity). As expected, a periodic sinusoidal-like pattern (Figure 10D) was obtained for proprioception sensing in all related neurons (only AS3 is presented for simplicity).

Finally, a collision test was carried out. While the animal was crawling, as induced by the CPG, a rigid obstacle was placed close to left side of the head. The stimulus received by neurons of different parts of the body was measured. As shown in the Figure 10E, the stimulus was not received by the neurons that are not located in the head (LUAL and LUAR). The neurons of the left part (BDUL) are stimulated more intensely.

## 4. Discussion

As analyzed in section 1, researches in the literature that try to simulate the *C. elegans*behavior responding to a certain stimulus use a model of a unique stimulus to activate a restricted neural network. On the contrary, in this paper we have focussed on modeling a wide repertoire of stimuli types, based on the main behavioral experiments carried out with the *C. elegans*worm (Hart, 2005).

Specifically, the following types of stimuli have been modeled:

- Direct touch, with a Gaussian-like force spread in the surrounding area.
- Plate tap, similar to direct touch but not localized in a specific area.
- Chemical quadrants and osmotic ring, considering the concentration at current position of the nematode.
- Static and dynamic drop tests, with Gaussian-like spread of the chemical concentration in the space.
- Global temperature transferred to all neurons of the worm.
- Different temperature gradients, taking into account the current position of the animal.
- Electric shocks, with periodic box functions.
- Light pulses, with box functions.
- Self shape sensing, with a simplification of the shape of the body.
- Muscle stretch sensing, considering the current length of each muscle.

Moreover, unlike other works that have been analyzed, this work takes the morphology of the sensory organs of *C. elegans*into consideration when transferring the stimuli signals to the sensory neurons of the worm.

The models have been implemented within the *Si elegans* platform, in which the user can simulate different kinds of behavioral experiments. Such platform has been used to test the correctness of the models. Nevertheless, stimuli modeling approach and its implementation presented in this paper may provide stimuli input for *C. elegans* behavioral experiments for various neuronal simulation systems, such as jLems (jLEMS, 2017) or pyLEMS (Vella et al., 2014).

The work has been focused only on the natural input that the neurons will receive during the simulation and not in the processes that convert such input into neural activation. The latter is out of the scope of this work and can be consulted e.g., in Nossenson and Messer (2010), where a model that generates neural activity spikes from the natural input is considered.

Future work includes modeling and implementation of natural inputs that are affected by the nematode itself. For instance, the concentration of a certain chemical in an area can be affected by the crawling of the animal in that area. Methods like Smooth Particle Hydrodynamics can be useful for these cases, but efficiency of the method has to be taken into account if close-to-real-time performance is desired.

## Author contributions

AM and PL defined the models described in this paper under the supervision of MO and GE. AM, PL, RÁ, and GE worked on the implementation. All authors contributed, reviewed and approved the final version of the manuscript.

### Conflict of interest statement

The authors declare that the research was conducted in the absence of any commercial or financial relationships that could be construed as a potential conflict of interest.
