# Supplementary material for: Modeling Behavioral Experiment Interaction and Environmental Stimuli for a Synthetic C. elegans
Source: Front Neuroinform. 2017 Dec 8;11:71. doi: 10.3389/fninf.2017.00071 (PMC5727351; doi:10.3389/fninf.2017.00071)
Supplement: Supplementary file 1 [file Table1.PDF]

Supplementary Material: Modelling Behavioural Experiment Interaction and Environmental Stimuli for a Synthetic C. elegans

1 APPENDIX A

Table S1. List of parameters that can be transferred to the neurons, the expected range of the values and their resolution.

Type	Sensory Input	Expected Range	Resolution
Chemosensation	Chemical Concentration	0 ... 200 M	1 μM
Thermosensation	Temperature	-100 ... 100 $°C$	0.01 $°C$
Mechanosensation	Force	0 ... 200 μN	0.01 μN
Galvanosensation	Current	0 ... 0.2 A	1 nA
Proprioception	Stretch	0 ... 3	0.0001
	Flexion	$-\pi$ ... π rad	0.0001 rad
Photosensation	Wavelength	250 ... 750 nm	0.0001 nm
	Light Intensity	0.01 ... 1.0 cd	0.0001 cd
