# Supplementary material for: Modeling Behavioral Experiment Interaction and Environmental Stimuli for a Synthetic C. elegans
Source: Front Neuroinform. 2017 Dec 8;11:71. doi: 10.3389/fninf.2017.00071 (PMC5727351; doi:10.3389/fninf.2017.00071)
Supplement: Supplementary file 2 [file Table2.PDF]

frontiers

Supplementary Material: Modelling Behavioural Experiment Interaction and Environmental Stimuli for a Synthetic C. elegans

1 APPENDIX B

Table S1. A list of recognized sensory neurons with corresponding types of sensory element and its position is annotated in the following. The position is annotated as the distance on the Anterior-to-Posterior axis, relative to the worm size (from 0 to 1), plus encoding of their lateral position (L = left, R = right, Dx = dorsal, Vx = ventral, Lx = lateral).

Neuron Group	Description of Associated Sensory Organ	Position
Chemosensation		
ADF	Amphid neuron	0 + L, R
ADL	Amphid neuron	0 + L, R
AQR	Ciliated endings exposed to the pseudocoelomic body fluid, projects into the ring	0.14 R
ASE	Amphid neurons, single ciliated endings	0 + L, R
ASG	Amphid neurons, single ciliated endings	0 + L, R
ASH	Amphid neurons, single ciliated endings	0 + L, R
ASI	Amphid neurons, single ciliated endings	0 + L, R
ASJ	Amphid neurons, single ciliated endings	0 + L, R
ASK	Amphid neurons, single ciliated endings	0 + L, R
AWA	Amphid wing cells	0.01 + L, R
AWB	Amphid wing cells	0.01 + L, R
AWC	Amphid wing cells	0.01 + L, R
BAG	Neuron, ciliated ending in head, no supporting cells, associated with ILso	0 + L, R
IL2	Inner labial neuron	0 + DL, DR, VL, VR, LL, LR
PHA	Phasmid neurons	0.95 + L, R
PHB	Phasmid neurons	0.95 + L, R
PQR	Directly exposed to the pseudocoelomic body fluid, projects into preanal ganglion	0.95 L
URX	Nonciliated dendritic endings in nose, Ring interneuron	0 + L, R
