# Supplementary material for: Modeling Behavioral Experiment Interaction and Environmental Stimuli for a Synthetic C. elegans
Source: Front Neuroinform. 2017 Dec 8;11:71. doi: 10.3389/fninf.2017.00071 (PMC5727351; doi:10.3389/fninf.2017.00071)
Supplement: Supplementary file 3 [file Table3.PDF]

Supplementary Material:
Article Title

1 APPENDIX B

Table S1. A list of recognized sensory neurons with corresponding types of sensory element and its position is annotated in the following. The position is annotated as the distance on the Anterior-to-Posterior axis, relative to the worm size (from 0 to 1), plus encoding of their lateral position (L = left, R = right, Dx = dorsal, Vx = ventral, Lx = lateral).

Neuron Group	Description of Associated Sensory Organ	Position
Thermosensation		
AFD	Amphid finger cell	0 + L, R
AWC	Amphid wing cells	0.01 + L, R
FLP	Branched, ciliated ending in head, no supporting cells, associated with ILso	0 + L, R
PHC	Neuron, striated rootlet in male, possibly sensory in tail spike	1 + L, R
PVD	Branched, lateral process adjacent to excretory canal	0.65 + L, R
Mechanosensation		
ADE	Ciliated anterior deirides, endings embedded within the cuticle	0,13 + L, R
ALM	Long processes, microtubule filled dendrites	anterior lateral body structure
AVM	Long processes, microtubule filled dendrites	anterior ventral body structure
CEP	Cephalic ciliated outer labial, endings embedded within the cuticle	0,01 + DL, DR, VL, VR
FLP	Brancehd neuron, ciliated ending in head, no supporting cells, associated with ILso	branched in head
IL1	Inner labial neuron with ciliated endings embedded within the cuticle	0 + DL, DR, VL, VR, LL, LR
OLL	Ciliated outer labial, endings embedded within the cuticle	0,01 + L, R
OLQ	Ciliated outer labial, endings embedded within the cuticle	0,01 + DL, DR, VL, VR
PDE	Ciliated posterior deirides, endings embedded within the cuticle	0,68 + L, R
PLM	Long processes, microtubule filled dendrites	tail, posterior lateral, ventrally directed
PVD	Branched neuron, lateral process adjacent to excretory canal	branched in body
PVM	Long processes, microtubule filled dendrites	posterior ventral body structure
