# Supplementary material for: Modeling Behavioral Experiment Interaction and Environmental Stimuli for a Synthetic C. elegans
Source: Front Neuroinform. 2017 Dec 8;11:71. doi: 10.3389/fninf.2017.00071 (PMC5727351; doi:10.3389/fninf.2017.00071)
Supplement: Supplementary file 4 [file Presentation1.PDF]

Supplementary Material: Modelling Behavioural Experiment Interaction and Environmental Stimuli for a Synthetic C. elegans

1 APPENDIX C

Because of *Si elegans* platform implementation reasons, the bell shaped ramps that model the touch events have been obtained with 2nd order polynomial functions:

$$F_p = \begin{cases} Acx^2 & \text{for } 0 \leq x \leq \frac{1}{4}t \\ A - Ac\left(x - 2\sqrt{\frac{1}{2c}}\right)^2 & \text{for } \frac{1}{4}t \leq x \leq \frac{1}{2}t \\ A & \text{for } \frac{1}{2}t \leq x \leq \frac{1}{2}t + T \\ A - Ac\left(x - T - 2\sqrt{\frac{1}{2c}}\right)^2 & \text{for } \frac{1}{2}t + T \leq x \leq \frac{3}{4}t + T \\ Ac\left(x - T - 4\sqrt{\frac{1}{2c}}\right)^2 & \text{for } \frac{3}{4}t + T \leq x \leq t + T \end{cases} \tag{S1}$$

In equation S1, F_p is the force transferred to the neurons, $t + T$ expresses the total duration of the stimuli (t specifying the ramp up and ramp down duration and T the touch duration, in ms) and A its amplitude (the inner parameter c is to be calculated from t). A considerably smaller ramp-up and ramp-down duration time is applied for the harsh stimulus, in comparison to the gentle touch.
